# Supplementary material for: Identifying repeat domains in large genomes
Source: Genome Biol. 2006 Jan 31;7(1):R7. doi: 10.1186/gb-2006-7-1-r7 (PMC1431705; doi:10.1186/gb-2006-7-1-r7)
Supplement: Additional File 1 — A zipped file of browsable HTML files with a complete list of the connected components in the repeat domain graph of human Repbase. [file gb-2006-7-1-r7-S1.gz › html/subgraphs/838.pdf]

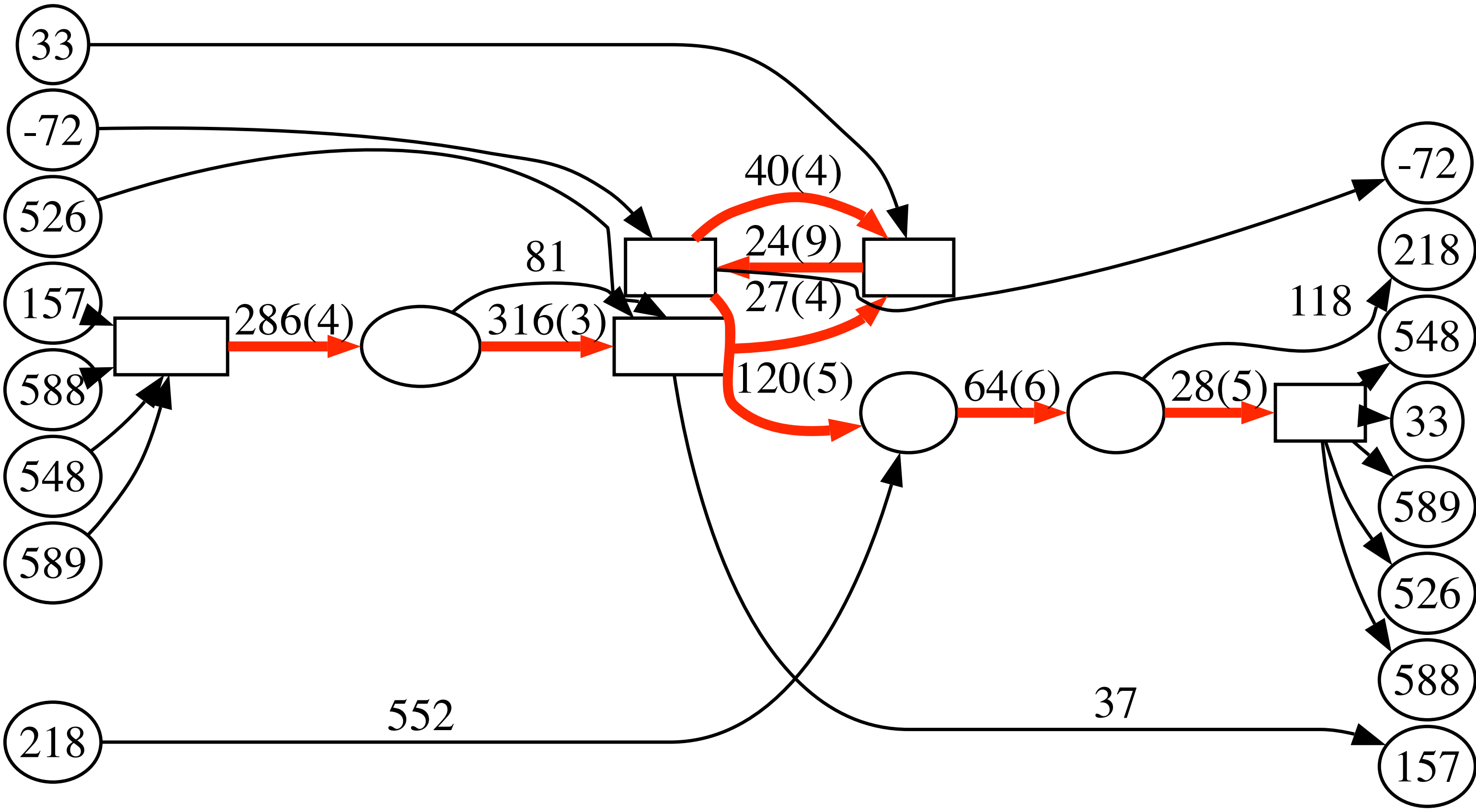
33
-72
526
157
588
548
589
218
286(4)
81
316(3)
40(4)
24(9)
27(4)
120(5)
64(6)
28(5)
118
552
37
-72
218
548
33
589
526
588
157
